# Ventral incisional hernia repair after liver transplant using posterior component separation with transversus abdominis release

**DOI:** 10.1007/s10029-026-03731-6

**Published:** 2026-05-26

**Authors:** Georgios Voidonikolas, Harriet G. Leibowitz, Justin A. Steggerda, Jill Frese, Todd Brennan, Steven A. Wisel, Tsuyoshi Todo, Nicholas Nissen, Ju Dong Yang, Philip S. Brazio, Irene Kim, Kambiz Kosari

**Affiliations:** Division of Transplant Surgery, Department of Surgery, Cedars- Sinai Medical Center, Los Angeles, CA USA

**Keywords:** Ventral incisional hernia, Posterior component separation, Transverse abdominus release, Mercedes-Benz incision

## Abstract

**Purpose:**

The optimal repair method for ventral incisional hernias (VIH) following orthotopic liver transplantation (OLT) has not been standardized. This study compares outcomes between use of open posterior component separation with transversus abdominis release (PCS-TAR) versus other hernia repair techniques (OHR) in patients with prior OLT from a single center.

**Methods:**

Patients with a prior OLT who underwent VIH repair along their trifurcation “Mercedes Benz” incision were identified at a single center between 2007 and 2022. The primary outcome of interest was incidence of hernia recurrence between patients who were treated with PCS-TAR versus OHR. Secondary outcomes included length of hospital stay (LOHS), surgical site complications (SSC), readmissions and reoperations. *P* values < 0.05 were considered significant.

**Results:**

Of 1,083 OLTs, there were 53 VIHs (4.9%) repaired, of which 23 (43%) underwent PCS-TAR and 30 (56%) underwent OHR. There were no statistical differences in the demographics between the groups including mean age (62.7 vs. 58.6 years, *p* = 0.08), male sex (69.6% vs. 73.3%, *p* = 0.52), and BMI > 30 kg/m^2^ (21.7% vs. 30%, *p* = 0.5). The median time from OLT to VIH repair was 96 weeks vs. 99.3 weeks (*p* = 0.35). Median follow-up was shorter in the PCS-TAR group (53 vs. 88.5 months, *p* < 0.01). The mean hernia width was similar in the two groups (10.98 vs. 11.88 cm, *p* = 0.71). VIH recurrence was 0% in PCS-TAR compared to 36.7% in OHR group (*p* < 0.01). The two groups had similar incidence of SSC and LOHS. Unplanned reoperations were seen only in the OHR group (0% vs. 13.3%, *p* = 0.03).

**Conclusions:**

PCS-TAR repair with mesh is a superior technique for VIH repair following OLT, offering a safe and effective approach with reduced hernia recurrence compared to other repair techniques in post-OLT patients.

## Introduction

Ventral incisional hernias (VIHs) constitute a frequent and resource-intensive long-term complication following orthotopic liver transplantation (OLT), with reported incidences ranging from 16.7% to 40% [1, 2]. In 2023, the field of liver transplantation surpassed a significant benchmark, with more than 10,000 OLTs performed—an 11.9% increase from the prior year and reflecting a sustained upward trend over the past decade [3]. Coupled with 5-year post-transplant survival rates exceeding 75%, the expanding population of liver transplant recipients has led to a growing burden of complex abdominal wall defects, necessitating increased operative intervention, multidisciplinary care, and utilization of healthcare resources [4–7].

In liver transplant recipients, VIHs commonly present as complex fascial defects involving one or more components of the bilateral subcostal incisions and the upper midline vertical extension of the standard Mercedes-Benz incision (MBI) used for transplantation. These hernias often involve the central aspect of the MBI and, not uncommonly, exhibit a component of loss of domain, further increasing the technical difficulty of repair. In addition, OLT recipients represent a uniquely high-risk population for postoperative VIH development, with contributory factors including male sex, age greater than 60 years, diabetes mellitus, obesity, advanced cirrhosis, immunosuppressive therapy—particularly corticosteroids and mammalian target of rapamycin inhibitors—as well as prior and post-OLT abdominal laparotomies and repeat liver transplantation [8].

Complex VIHs often necessitate advanced reconstructive approaches; accordingly, a broad spectrum of repair strategies has been described over the past several decades, encompassing both open and minimally invasive techniques. Further expanding the available options, multiple mesh placement planes have been utilized, including intraperitoneal (underlay), preperitoneal or retromuscular (sublay), bridging (inlay), and premuscular (onlay) configurations [9]. The posterior component separation with transversus abdominis release (PCS-TAR) has emerged as a favored technique for the repair of complex abdominal wall defects, in part because of reported low recurrence rates of approximately 4.7% [10–12]. Fundamental principles of the PCS-TAR technique include wide mesh overlap and preservation of the abdominal neurovascular bundles to facilitate durable reconstruction of large defects. However, data regarding the application of PCS-TAR for VIH repair in OLT recipients remains limited, and when performed through a new midline incision in this population, recurrence rates as high as 25% have been reported [13].

Beginning in mid-2018, our transplant center restructured its approach to VIH repair in OLT recipients, and four transplant surgeons underwent training in open PCS-TAR technique using the preexisting subcostal component of the MBI. Following this transition, patients requiring VIH repair were progressively referred to and managed by these surgeons, and over time, all eligible cases were preferentially assigned to this group.

The objective of this study is to evaluate the safety and efficacy of this modified PCS-TAR approach in post-OLT patients with incisional hernias, and to compare outcomes with those of other hernia repair methods (OHR) previously employed at our institution.

## Methods

### Study design and patient selection

After Institutional Review Board approval, a retrospective review was conducted of all OLT recipients who underwent VIH repair at our institution between November 1, 2007 and November 30, 2022. Patients were stratified into two groups based on technique of VIH repair: (1) the PCS-TAR group, consisting of patients who underwent VIH repair using the PCS-TAR technique performed by four transplant surgeons between 2018 and 2022, with data collected prospectively and entered in real time by the operating surgeons; and (2) the OHR group, consisting of patients who underwent VIH repair prior to 2022 using various alternative techniques, performed by fourteen surgeons, with data collected retrospectively as a historic control. Other techniques for VIH repair included primary repair, open or laparoscopic underlay/intraperitoneal repair with mesh, or onlay mesh repair.

All surgical procedures were conducted at Cedars-Sinai Medical Center (CSMC), Los Angeles, California, USA. Emergency hernia repairs were excluded.

### Data collection and outcomes

Patient demographics, hernia characteristics, operative details, and postoperative outcomes were abstracted from the electronic medical record. Post-OLT, patients were followed in the transplant clinic every 3 months during the first year and every 6 months thereafter, according to our standard post-OLT protocol. Hernia recurrence was assessed using a physical examination and radiographic imaging when clinically indicated, most commonly with computed tomography (CT).

The primary outcome was hernia recurrence, as determined by clinical examination and/or cross-sectional imaging including computed tomography or magnetic resonance imaging. Secondary outcomes included length of hospital stay (LOHS), 30-day surgical site infections (SSIs)-categorized as superficial, deep, or mesh-related- and 30-day surgical site occurrences (SSOs) including SSI, seroma, and hematoma. Any SSI or SSO requiring procedural intervention, such as wound opening or debridement, percutaneous drainage or suture or mesh excision, was classified as a surgical site occurrence requiring procedural intervention (SSOPI). Unplanned reoperations and hospital readmissions within one year of repair were also recorded.

### PCS-TAR surgical technique

**Fig. 1:**
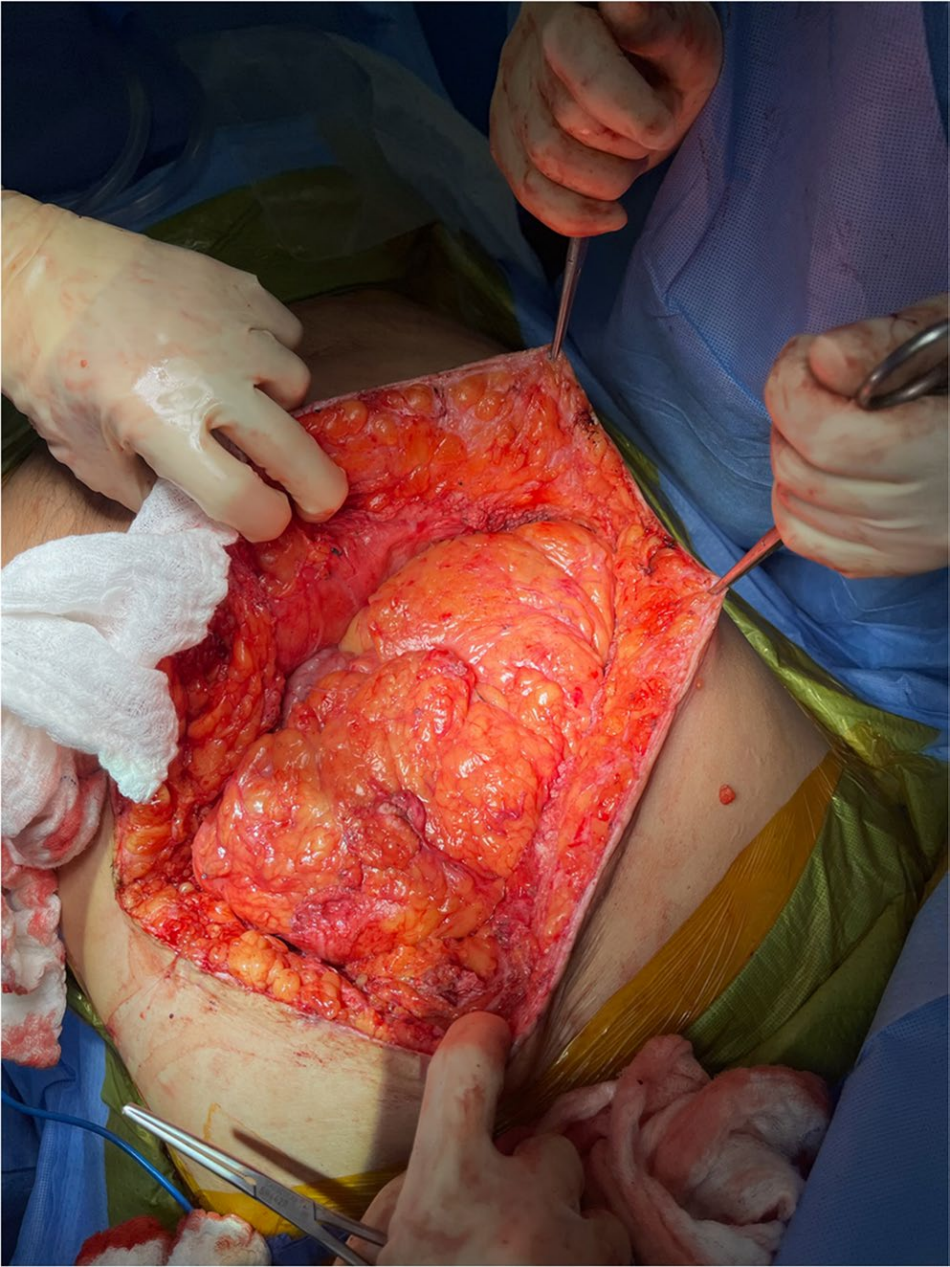
Access to the abdominal cavity is obtained through the subcostal component of the preexisting Mercedes-Benz incision. (Patient’s head oriented toward the bottom of the image)

Abdominal access is obtained through the subcostal component of the existing MBI. Adhesiolysis is performed as needed to allow reduction of hernia contents and delineation of the fascial edges (Fig. 1).

**Fig. 2:**
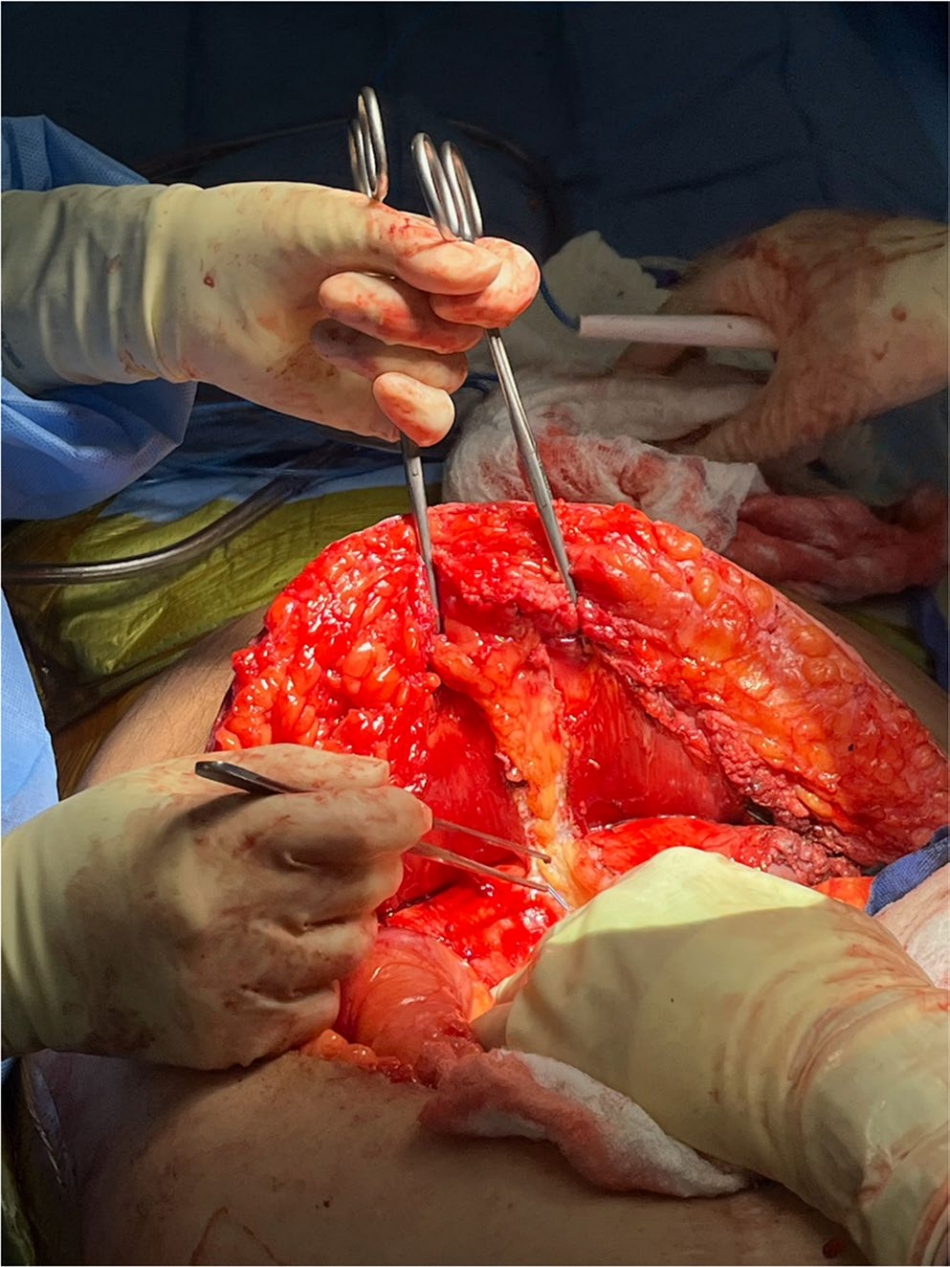
At the cranial and caudal corners, the medial edge of the posterior fascia is transected and “dropped” and the linea alba is not separated. (Patient’s head oriented toward the bottom of the image)

The retrorectus space is developed on both sides of midline. The posterior rectus sheath is dissected free and incised medially, lateral to its junction with the linea alba, which is preserved (Fig. 2). As described by Novitski, et al. [14], the posterior rectus sheath is incised 5 mm medial to the linea semilunaris. The posterior lamella and the transversus abdominis are released and further preperitoneal plane is created while avoiding and preserving the neurovascular bundles.

Caudally, the dissection extends as low as needed, including the Retzius space as described elsewhere [15–17]. If there is an accompanying umbilical hernia, dissection is extended past the umbilicus, the hernia sac is removed from the defect, and the anterior sheath defect is suture repaired from the inside. The cranial dissection extends into the retro-xiphoid space and to the costal margins bilaterally.

**Fig. 3:**
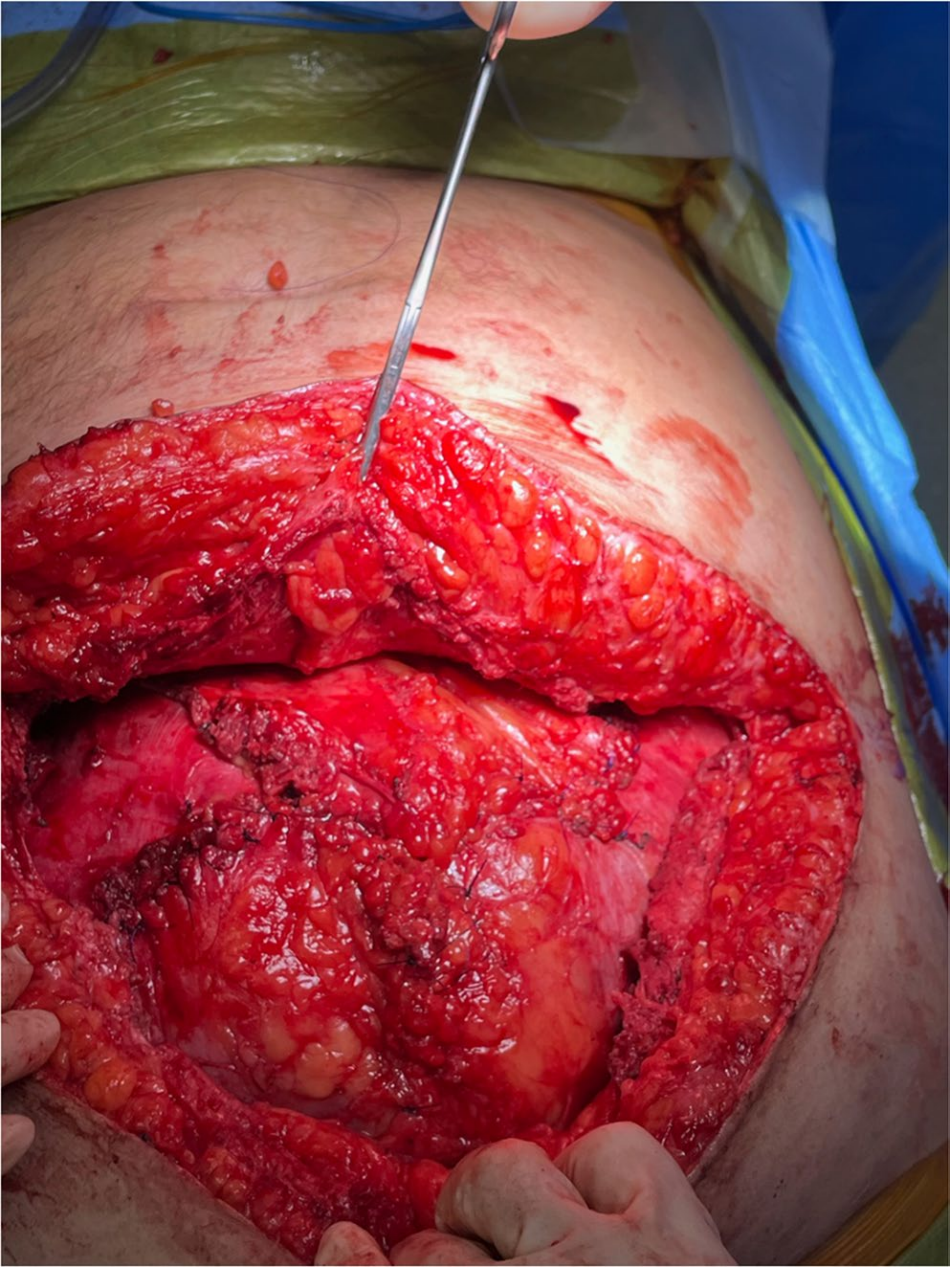
Reconstruction of the posterior layer during PCS-TAR. Following posterior dissection, four mobilized posterior fascial–peritoneal flaps are approximated and sutured in an intersecting, cruciform configuration. This orthogonal closure recreates a continuous posterior layer, re-establishes the retromuscular/preperitoneal pocket, and isolates the mesh plane from the intra-abdominal contents. (Patient’s head oriented toward the bottom of the image)

Following completion of the posterior dissection, four posterior-layer flaps are present and are closed with several running and figure-of-eight 3 − 0 PDS sutures, creating a cruciform configuration (Fig. 3). If this closure is not possible, the attached peritoneum is utilized to properly isolate the plane from the underlying abdominal cavity. Alternatively, a patch of omentum or a small piece of Vicryl or biologic mesh can be used. An adequate space of at least 4 cm beyond the fascial defect is created in the craniocaudal and lateral planes.

**Fig. 4:**
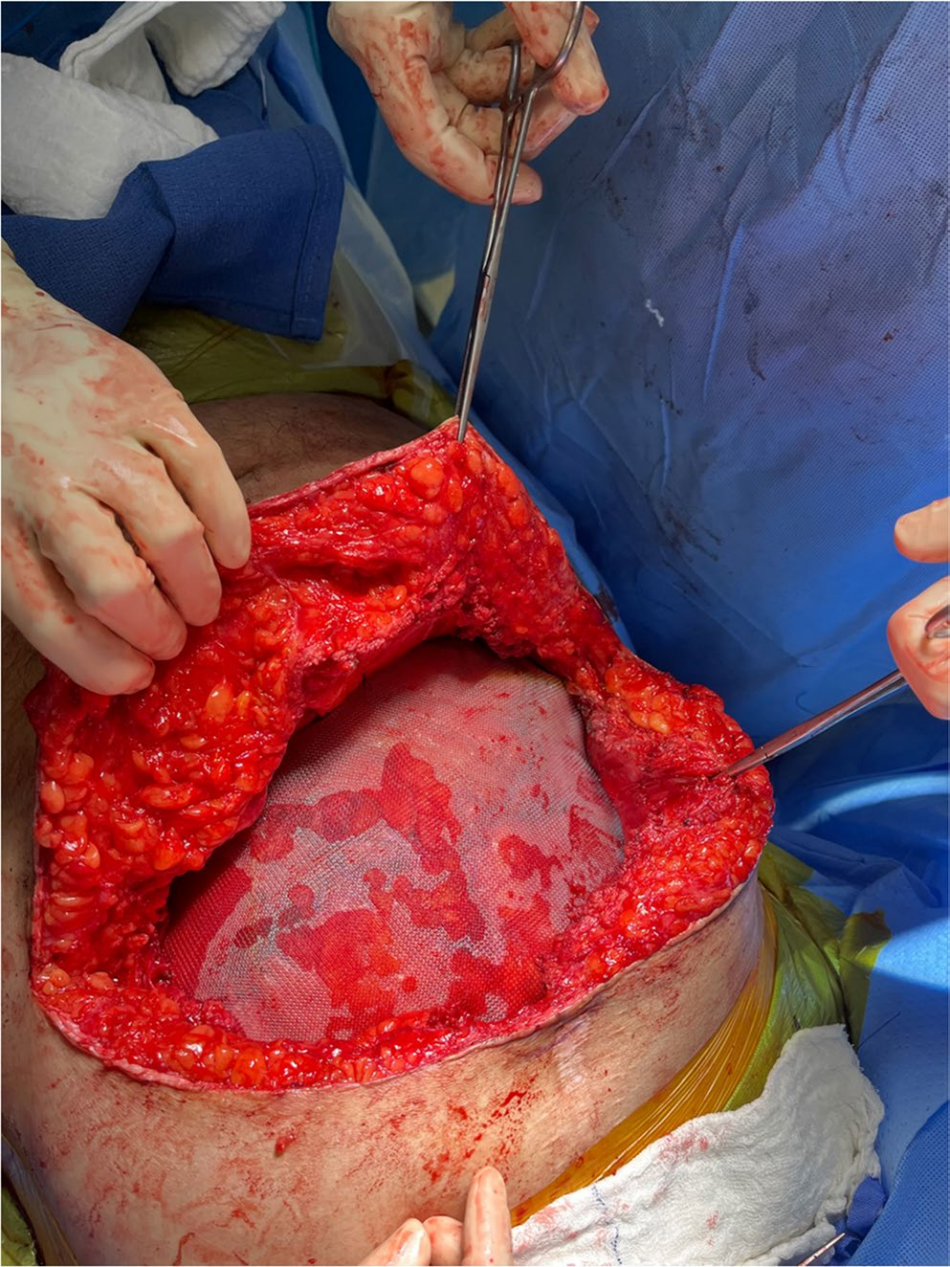
A medium-weight Polypropylene mesh is placed in the retromuscular/preperitoneal space (Patient’s head oriented toward the bottom of the image)

A single-piece, medium-weight, custom-fitted polypropylene mesh is placed widely in the retromuscular/preperitoneal space (Fig. 4). The mesh is anchored on the posterior fascia with a few interrupted 3 − 0 PDS sutures. No fixation of mesh to the costal margin or bone is performed, and care is taken that the mesh is flat-fitted without folds.

**Fig. 5:**
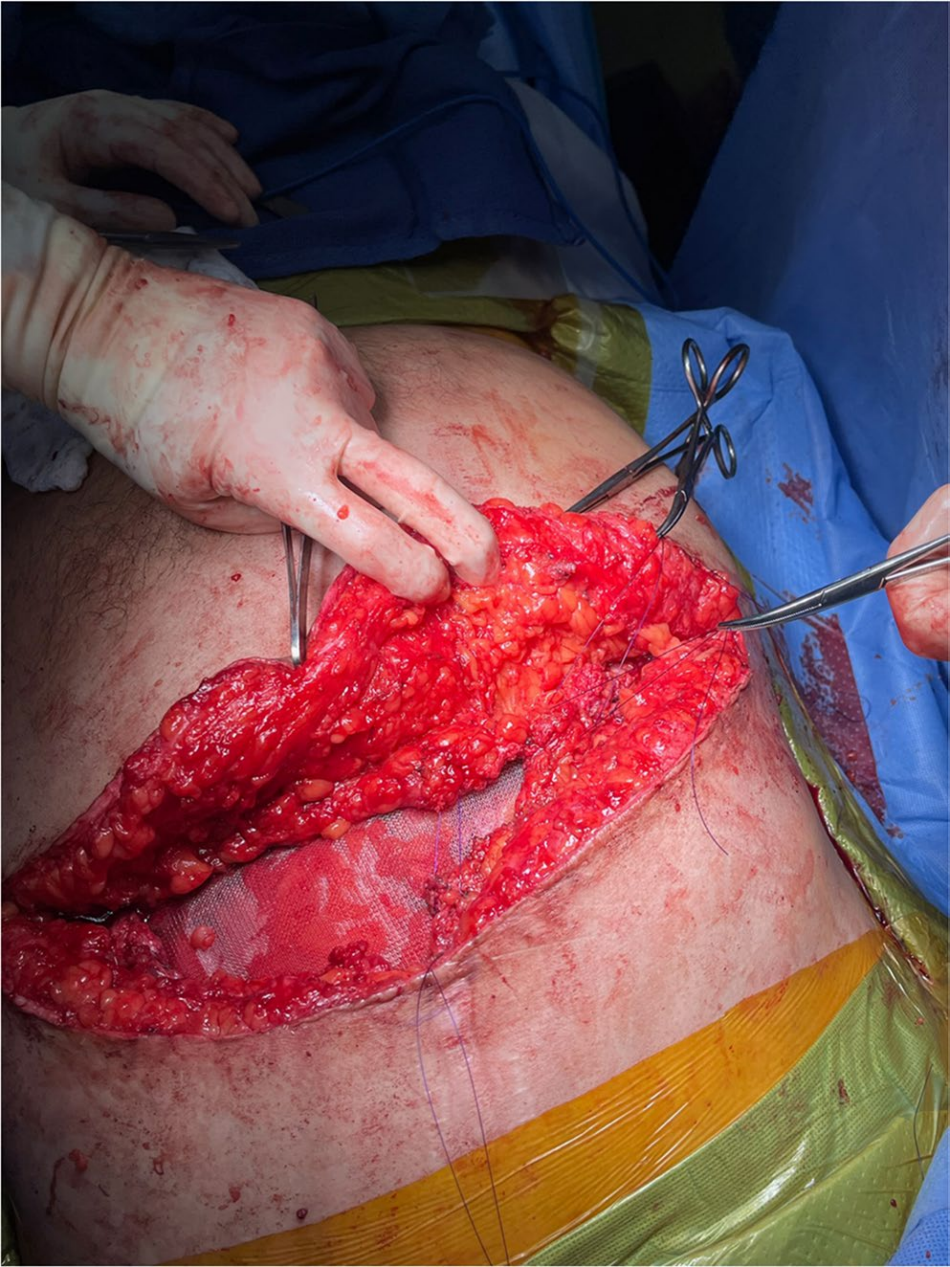
The anterior sheath and fascia are closed, when possible, with running and figure-of-eight 2 − 0 PDS sutures, usually in a transverse direction. (Patient’s head oriented toward the bottom of the image)

The anterior fascia edges are then closed with interrupted figure-of-eight 2 − 0 PDS sutures (Fig. 5). The mesh is incorporated into this closure with one of the two throws of each of the figure-of-eight sutures catching a small portion of it. If, due to loss of domain, the anterior fascia cannot close, the free edges are circumferentially sewn onto the mesh, followed by sewing the edges of the subcutaneous fat onto the exposed mesh as an attempt to reduce future seromas. The subcutaneous space is closed in layers and the excessive skin and previous scar are excised. A compressive dressing and an abdominal binder are applied. All attempts are made to extubate patients deep and avoid sudden Valsalva events.

### Statistics

Descriptive statistics are reported as measures of central tendency and variability, including the median with interquartile range (IQR) and the mean with standard deviation (SD), as appropriate. Continuous variables were compared using Mann-Whitney U test, while categorical variables were analyzed using the chi-square test or Fisher’s exact test where appropriate. Two-sided *P* values < 0.05 were considered significant. Multivariable analysis was performed to evaluate the association between surgical technique and hernia recurrence. Given the small sample size and the absence of recurrence events in the PCS-TAR group, standard logistic regression was not appropriate due to complete separation. Therefore, a Firth penalized logistic regression model was used to reduce small-sample bias and provide stable estimates. Time-to-event analysis for hernia recurrence was performed using Kaplan–Meier methodology, with differences between groups assessed using the log-rank test. All statistical analyses were performed using SPSS for Windows (version 22.0; IBM Corp., Armonk, NY).

## Results

Among 1083 OLTs performed between 2007 and 2022, 53 patients underwent VIH repair, of whom 23 (43%) were treated with PCS-TAR and 30 (57%) underwent OHR. Patient demographics and baseline characteristics are summarized in Table 1.

**Table 1:** Patient demographics and comorbidities of the PCS-TAR group (*n* = 23) and the OHR group (*n* = 30)

	*n* (%)	*n* (%)	*P*-Value
Mean age, years at time of hernia repair, [± SD]	62.7 [± 8.9]	58.6 [± 9.7]	0.08
*Gender*			0.52
Male	16 (69.6)	22 (73.3)	
Female	7 (30.4)	8 (26.7)	
Median BMI, kg/m² at time of hernia repair, [IQR]	27.6 [25.7–31.5]	28.3 [25–30.4.4]	0.64
Median weeks between OLT and IH repair, [IQR]	96.0 [53.7–134.7.7.7]	99.3 [68.6–176.3.6.3]	0.35
Obesity (BMI ≥ 30)	5 (21.7)	9 (30.0)	0.5
Diabetes	4 (17.4)	11 (36.7)	0.14
Coronary artery disease	2 (8.7)	3 (10.0)	0.91
Arrythmia	2 (8.7)	2 (6.7)	0.75
Chronic obstructive pulmonary disease	3 (13.0)	1 (3.3)	0.17
CKD	4 (17)	5 (16)	0.26
Hx smoking	3 (13.0)	4 (13.3)	0.92
Post-transplant lymphoproliferative disorder	0	1 (3.3)	0.46
Median MELD score at OLT, [IQR]	32.7 [28–40]	33 [25–40]	0.49
*Type of immunosuppression*			
Tacrolimus	23 (100)	30 (100)	1
Mycophenolate Mofetil	10 (38.5)	11 (36.7)	0.15
Prednisone	21 (91.3)	20 (87.0)	0.29
Mean ASA score	2.73	2.87	0.34
ASA ≥ 3	17(74)	18 (60)	0.47

Continuous variables are reported as mean ± standard deviation or median with interquartile range (IQR), as appropriate, and were compared using the Mann–Whitney U test. Categorical variables are presented as number (percentage) and were analyzed using the chi-square test or Fisher’s exact test, as appropriate. A two-sided *P* value < 0.05 was considered statistically significant

*PCS-TAR* = Posterior Component Separation with Transversus Abdominis Release, *OHR* = Other Hernia Repair, *OLT* = orthotopic liver transplant, *IH* = Incisional Hernia, *BMI* = Body Mass Index, *CKD* = Chronic Kidney Disease, *MELD* = Model for End-Stage Liver Disease, *ASA* = American Society of Anesthesiologists

There were no statistical differences in demographics between study cohorts. Mean age at the time of hernia repair was 62.7 ± 8.9 years in the PCS-TAR group and 58.6 ± 9.7 years in the OHR group (*P* = 0.08). Most patients were male in both groups (69.6% vs. 73.3%, *P* = 0.52). Median body mass index (BMI) was 27.6 kg/m^2^ (IQR 25.7–31.5) in the PCS-TAR group and 28.3 kg/m^2^ (IQR 25.0–30.4.0.4) in the OHR group (*P* = 0.64).

The median interval between liver transplantation and hernia repair was similar between groups (96.0 weeks [IQR 53.7–134.7.7.7] vs. 99.3 weeks [IQR 68.6–176.3.6.3], *P* = 0.35). Common comorbidities included obesity (BMI ≥ 30 kg/m^2^), diabetes mellitus, chronic kidney disease, coronary artery disease, arrhythmia and chronic obstructive pulmonary disease, with no statistically significant differences between groups.

Hernia characteristics and operative details are summarized in Table 2. Mean hernia width was similar between groups (10.98 ± 5.0 cm in the PCS-TAR group vs. 11.88 ± 6.6 cm in the OHR group, *P* = 0.71). Mean hernia area was not statistically different (85.0 ± 71.7 cm² vs. 140.1 ± 137.2 cm², *P* = 0.21). All hernias originated from a prior MBI, and all cases were classified as clean wounds at the time of repair.

**Table 2:** Hernia characteristics and operative details of the PCS-TAR group (*n* = 23) and the OHR group (*n* = 30)

Variables	*n* (%)	*n* (%)	*P*-Value
Mean Hernia width, cm, [± SD]	10.98 [± 5.0]	11.88 [± 6.6]	0.71
Mean Hernia area, cm², [± SD]	85.0 [± 71.7]	140.1 [± 137.2]	0.21
*Original incision for liver transplant*			
Mercedes Benz	23 (100.0)	30 (100.0)	1
*Component of defect*			0.07
Whole MB incision	10 (43.5)	6 (20)	
Lateral component	17 (73.9)	23 (77)	
Midline component	17 (73.9)	21 (70)	
*CDC wound class*			
Clean	23 (100)	30 (100)	1
Mean Mesh area, cm², [± SD]	253.5 [± 190.65]	370.8 [± 280.1]	0.15
*Mesh type*			
Synthetic	23(100)	5(16.7)	< 0.01
Synthetic with barrier or dual layered	0	16 (53.4)	
Biologic	0	6 (20)	
None	0	3 (10.0)	
*Repair type*			
Underlay/intraperitoneal with mesh	0	19 (63.3)	< 0.01
Sublay/retroperitoneal-retromuscular with mesh	23 (100)	3 (10.0)	
Onlay with mesh	0	5 (16.6)	
Primary	0	2 (6.7)	
Complete Anterior Fascial Closure	20 (87.0)	21 (70.0)	0.28
Placement of drain	1 (4.4)	8 (27.6)	0.03

Continuous variables are reported as mean ± standard deviation and were compared using the Mann–Whitney U test. Categorical variables are presented as number (percentage) and were analyzed using the chi-square test or Fisher’s exact test, as appropriate. A two-sided *P* value < 0.05 was considered statistically significant

*PCS-TAR* = Posterior Component Separation with Transversus Abdominis Release, *OHR* = Other Hernia Repair, *CDC* = Centers for Disease Control and Prevention

All PCS-TAR repairs were performed using medium-weight polypropylene mesh placed in a sublay/retrorectus position. In contrast, the OHR group demonstrated significant heterogeneity in both mesh selection and repair technique (*P* < 0.01 for both). Complete anterior fascial closure was achieved in 20 patients (87.0%) in the PCS-TAR group and 21 patients (70.0%) in the OHR group (*P* = 0.28). Drain placement was less frequent in the PCS-TAR group compared with the OHR group (4.4% vs. 27.6%, *P* = 0.03).

Postoperative outcomes are summarized in Table 3. Median follow-up duration was significantly shorter in the PCS-TAR group compared with the OHR group (53 months [IQR 48–65] vs. 88.5 months [IQR 51–125], *P* < 0.01).

**Table 3:** Postoperative outcomes for the PCS-TAR group (*n* = 23) and the OHR group (*n* = 30). Continuous variables are reported as mean ± standard deviation or median with interquartile range (IQR), as appropriate, and were compared using the Mann–Whitney U test. Categorical variables are presented as number (percentage) and were analyzed using the chi-square test or Fisher’s exact test, as appropriate. A two-sided *P* value < 0.05 was considered statistically significant

	*n* (%)	*n* (%)	*P*-Value
Median follow-up, months [IQR]	53 [48–65]	88.5 [51–125]	< 0.01
Mean LOHS, days, [± SD]	3.7 [± 2.1]	4.2 [± 3.9]	0.86
*30-day SSI*	2 (8.7%)	4 (13.3%)	0.69
Superficial	2 (8.7%)	1 (3.3%)	0.57
Deep	0 (0.0%)	3 (10.0%)	0.25
Infected synthetic mesh	0 (0.0%)	2 (6.7%)	0.5
*30-day SSO (excludes SSI)*	3 (13.0)	4 (13.3)	0.97
Seroma	2 (8.7)	3 (10.0)	0.87
Hematoma	1 (4.3)	1 (3.3)	0.84
*30-day SSOPI*	3 (13)	6 (20.0)	0.5
Wound opening (for SSI)	1 (4.3)	3 (10.0)	0.62
Wound debridement (for SSI)	0	1 (3.3)	0.38
Percutaneous drainage or aspiration	2 (8.7)	3 (10.0)	0.87
*Medical complications*	2 (8.7)	3 (10.0)	0.87
*Unplanned Reoperation Required*	0	4 (13.3)	0.03
#Unplanned reoperations	0	7	
*Hernia recurrence*	0	11 (36.7)	< 0.01
*Readmission Required*	1 (4.3)	4 (13.3)	0.25
# Readmissions	1	10	

*PCS-TAR* = Posterior Component Separation with Transversus Abdominis Release, *OHR* = Other Hernia Repair, *LOHS* = Length of Hospital Stay, *SSI* = Surgical Site Infection, *SSO* = Surgical Site Occurrence, *SSOPI* = Surgical Site Occurrence Requiring Procedural Intervention

The primary outcome of VIH recurrence occurred in 11 patients (36.7%) in the OHR group and in no patients (0%) in the PCS-TAR group (*P* < 0.01). Among patients who experienced recurrence, the median time to recurrence was 29 months [IQR 18–76].

**Fig. 6:**
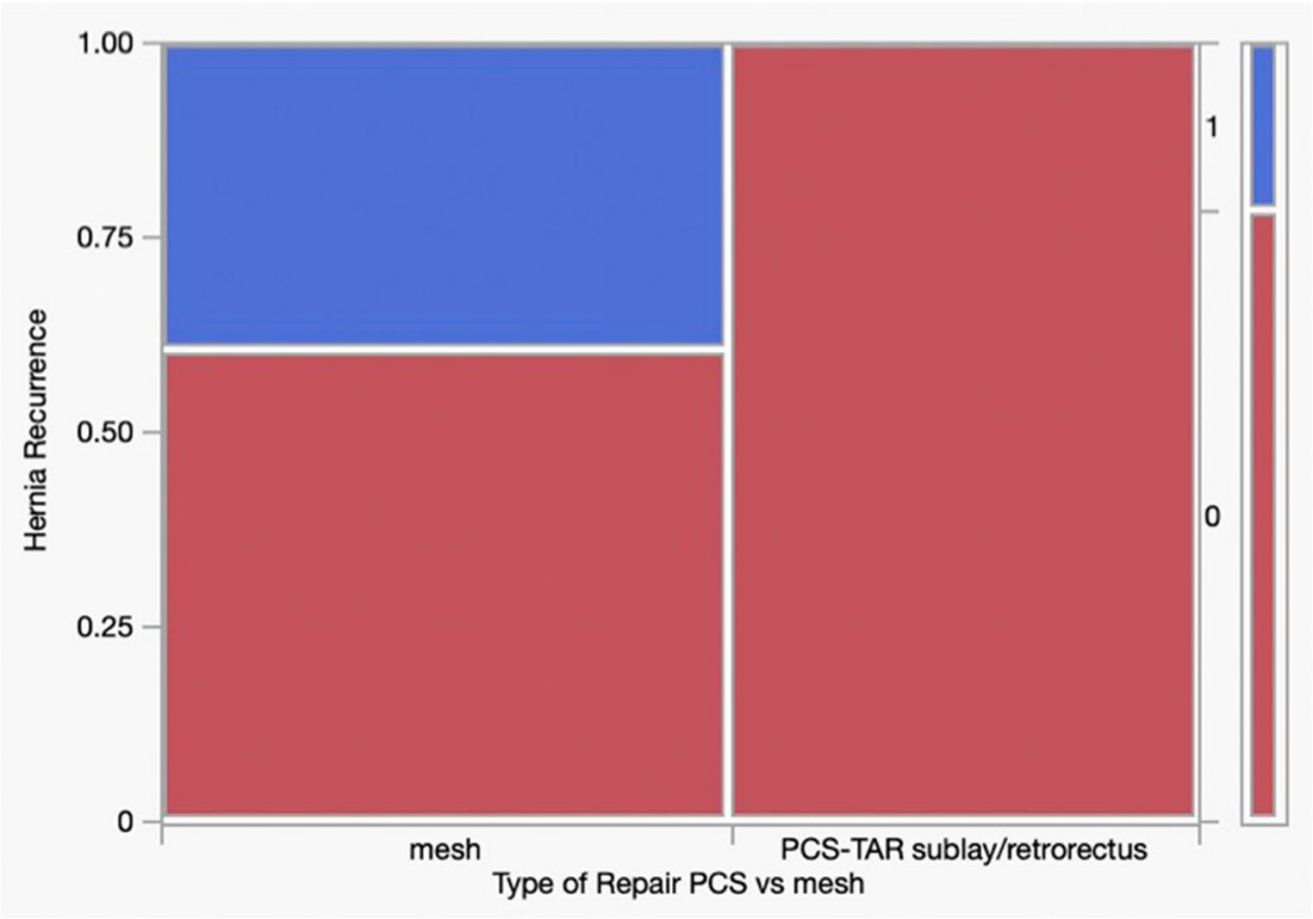
Subgroup Analysis of Mesh-Based Repairs. Comparison of hernia recurrence between PCS-TAR and mesh-based OHR (excluding primary suture repairs). Recurrence was significantly lower in the PCS-TAR group (0/23 [0%]) compared to mesh-based OHR (10/27 [37.0%], *p* < 0.001)

**Fig. 7:**
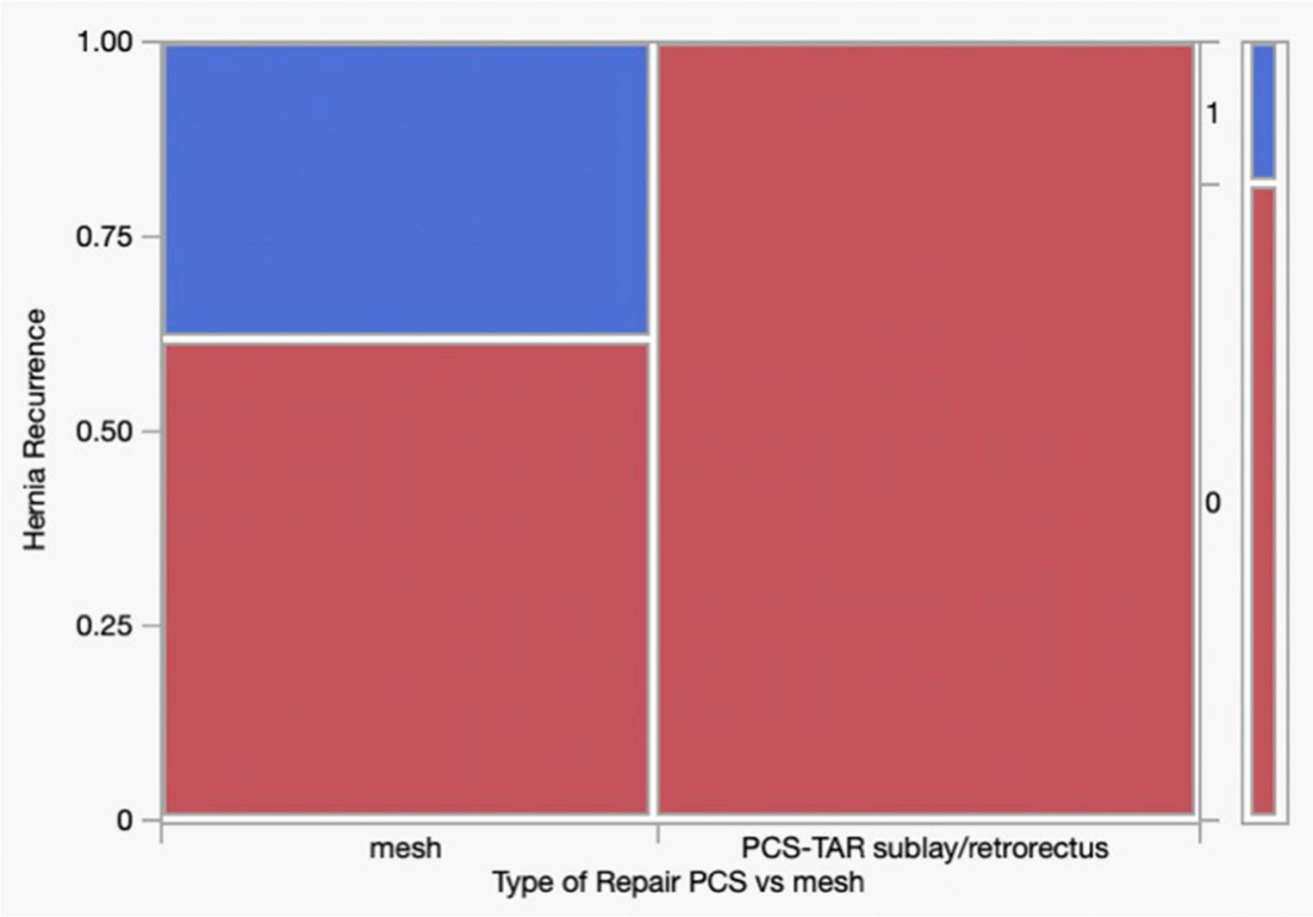
Subgroup Analysis of Sublay/Retromuscular Repairs. Comparison of hernia recurrence between PCS-TAR and sublay/retromuscular OHR (excluding primary, onlay, and bridging repairs). Recurrence was significantly lower in the PCS-TAR group (0/23 [0%]) compared to sublay/retromuscular OHR (8/21 [38.1%], *p* = 0.001)

A detailed breakdown of repair techniques and incidences of recurrence and complications within the OHR cohort is provided in Table 4. To address heterogeneity within the OHR group, subgroup analyses were performed. When restricted to mesh-based repairs (excluding primary suture repair), recurrence remained significantly lower in the PCS-TAR group (0/23 (0%) vs. 10/27 (37.0%), *p* < 0.001) (Fig. 6). A second subgroup analysis further excluding onlay and bridging repairs and limited to sublay/retromuscular repairs similarly demonstrated lower recurrence in the PCS-TAR group (0/23 [0%] vs. 8/21 [38.1%], *p* = 0.001) (Fig. 7).

**Table 4:** Operative and clinical characteristics of patients with hernia recurrence in the OHR cohort. Categorical variables are presented descriptively. Data shown include repair technique, mesh type, operating surgeon, duration of follow-up, time to recurrence, method of recurrence detection, and reoperation details among patients in the OHR group who experienced hernia recurrence. Abbreviations: OHR = Other Hernia Repair; IPOM = Laparoscopic Intraperitoneal Onlay Mesh; CT = Computed Tomography; US = Ultrasound; SBO = Small Bowel Obstruction

OHR Technique	Mesh Type	Recurrance	Time from Initial Repair to Recurrance (months)	Recurrance Detection Method	Reoperation	Reason for Reoperation
Laproscopic Underlay (IPOM)	Gore Dual	1	24	Clinic Visit + CT	0	N/A
Open Underlay	Composix Dual	1	81	Clinic Visit	0	N/A
Open Underlay	Composix Dual	1	8	Clinic Visit + US	0	N/A
Open Underlay	Supramesh	1	29	Clinic Visit + CT	2	2 Additional Operations for Recurrence + SBO
Open Underlay	Gore Dual	1	11	Clinic Visit + CT	0	N/A
Open Underlay	Flex HD	1	45	Clinic Visit + CT	0	N/A
Open Underlay	Symbotex	1	18	Clinic Visit + CT	0	N/A
Open Underlay	Supramesh	0	N/A	N/A	1	Mesh Excision for Infection
Onlay	Polypropylene	1	91	Clinic Visit + CT	3	(1) Post-op Hematoma (2) Post-op SBO (3) Recurrence/SBO
Sublay/Retroperitoneal	Polypropylene	1	76	Clinic Visit	0	N/A
Primary	N/A	1	22	Clinic Visit	0	N/A
Primary + Birectus Advancement	Strattice	1	5	Unknown - Outside Hospital	1	Mesh Excision for Infection

To account for potential confounding and the limitations of univariate analysis, a multivariable model was constructed. On multivariable Firth penalized logistic regression analysis, treatment with PCS-TAR was associated with significantly lower odds of hernia recurrence compared with OHR (B = − 2.758, SE = 1.162, *p* = 0.004). None of the other covariates were significantly associated with recurrence in the adjusted model (all *p* > 0.05) (Table 5).

**Table 5:** Firth penalized logistic regression to estimate the association between treatment condition and hernia recurrence while adjusting for demographic and clinical covariates. Coefficients (B) are reported on the log-odds scale. Wald chi-square statistics and associated p-values are reported for hypothesis testing. The Firth correction was applied to reduce small-sample bias and mitigate issues of separation. Abbreviations: BMI = Body Mass Index; DM = Diabetes Mellitus; CAD = Coronary Artery Disease; COPD = Chronic Obstructive Pulmonary Disease; CKD = Chronic Kidney Disease

Predictor	B	SE	Wald χ²	*P*-Value
Intercept	−0.517	0.806	0.367	0.545
**Treatment (0 = OHR; 1 = PSC-TAR)**	**−2.758**	**1.162**	**8.27**	**0.004**
Gender (0 = Female; 1 = Male)	−0.394	0.77	0.227	0.633
Age (0 = ≤ 60; 1 = > 60)	−0.703	0.728	0.798	0.372
BMI > 30 (0 = No; 1 = Yes)	−0.132	0.817	0.023	0.879
Smoking History (0 = No; 1 = Yes)	1.241	1.057	1.161	0.281
DM (0 = No; 1 = Yes)	0.716	0.778	0.764	0.382
CAD (0 = No; 1 = Yes)	0.312	1.093	0.073	0.787
COPD (0 = No; 1 = Yes)	1.143	1.505	0.415	0.519
CKD (0 = No; 1 = Yes)	0.373	0.777	0.193	0.66

**Fig. 8:**
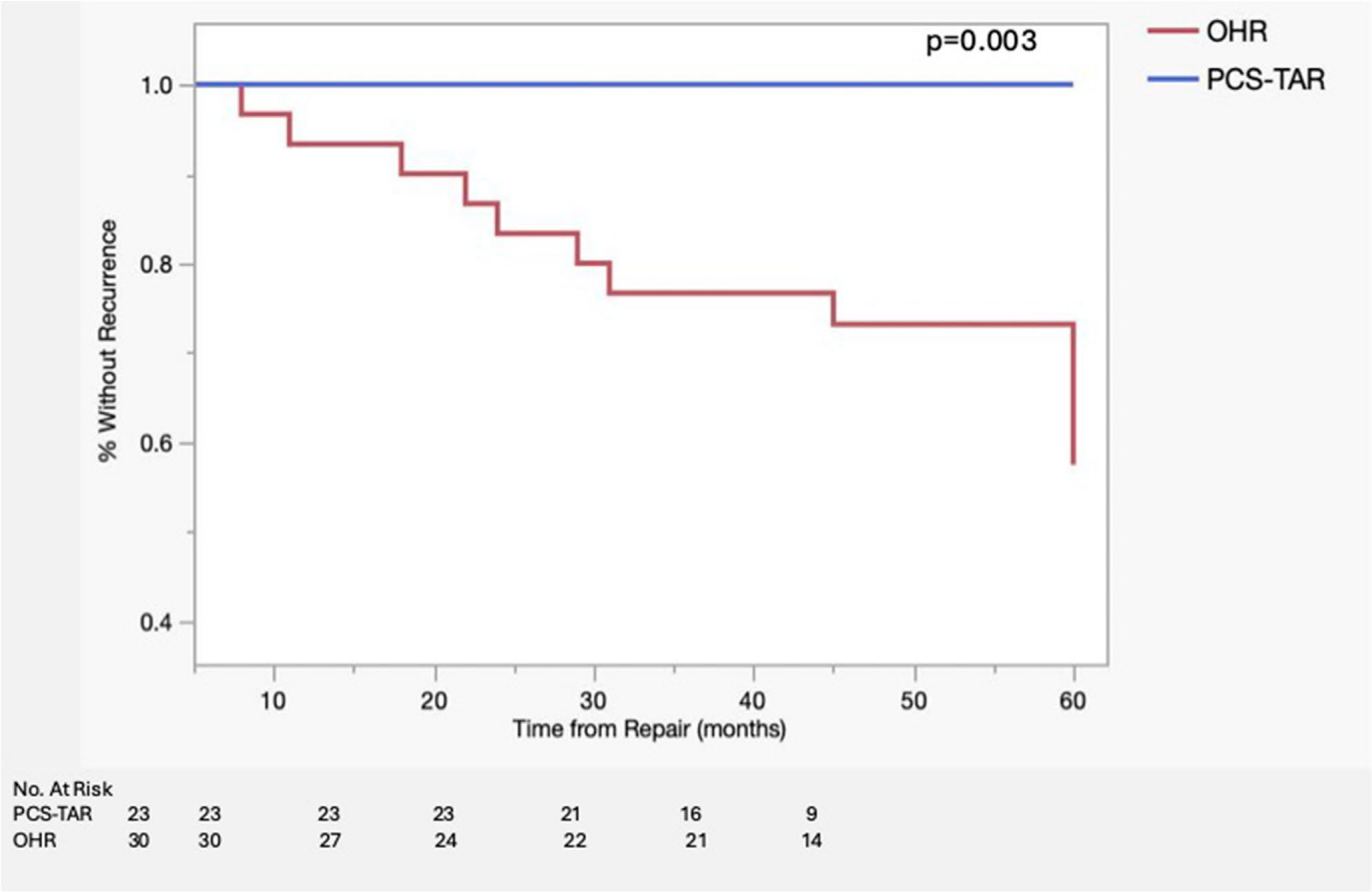
Kaplan–Meier Analysis of Recurrence-Free Survival. Kaplan–Meier curve demonstrating recurrence-free survival following ventral incisional hernia repair in PCS-TAR and OHR groups. Events represent hernia recurrence. Recurrence-free survival was significantly higher in the PCS-TAR group (log-rank *p* = 0.003)

Kaplan–Meier analysis of time to hernia recurrence demonstrated significantly improved recurrence-free survival in the PCS-TAR group compared to OHR (log-rank *p* = 0.003) (Fig. 8).

There were no significant differences between groups in LOHS (3.7 ± 2.1 vs. 4.2 ± 3.9 days, *P* = 0.86) or overall medical complications (8.7% vs. 10.0%, *P* = 0.87). Rates of SSI, SSO and 30 day SSOPI were also similar between groups.

No patients in the PCS-TAR group required unplanned reoperation, whereas four patients (13.3%) in the OHR group underwent a total of seven unplanned reoperations (*P* = 0.03). Hospital readmission occurred in one patient (4.3%) in the PCS-TAR group and in four patients (13.3%) in the OHR group, a difference that was not statistically significant (*P* = 0.25).

## Discussion

In this single-center cohort of liver transplant recipients undergoing ventral incisional hernia repair, PCS-TAR performed through the preexisting transplant incision was associated with a markedly lower rate of hernia recurrence compared with other repair techniques. Notably, no recurrences were observed in the PCS-TAR group during a median follow-up of 53 months, whereas more than one-third of patients undergoing alternative repair strategies experienced recurrence. Although recurrence may emerge with longer follow-up or larger cohorts, these findings suggest that the PCS-TAR approach offers a durable repair with low recurrence risk in a uniquely challenging patient population.

Importantly, baseline patient characteristics, comorbidities, hernia size, and wound classification were similar between groups, indicating that differences in outcomes were more likely attributable to operative strategy rather than patient selection. While median follow-up was longer in the OHR group, the median time to recurrence was 29 months, a time frame captured within the follow-up period of both cohorts, supporting the robustness of the observed recurrence difference. Furthermore, postoperative morbidity—including length of stay, surgical site complications, medical complications, and readmissions—was comparable between groups, while unplanned reoperations occurred significantly less frequently following PCS-TAR, an outcome of particular relevance in immunosuppressed transplant recipients.

These findings were consistent across multiple analytical approaches. Multivariable analysis using a Firth penalized logistic regression model demonstrated that PCS-TAR was independently associated with lower odds of hernia recurrence, despite the small sample size and absence of recurrence events in the PCS-TAR group. In addition, time-to-event analysis using Kaplan–Meier methodology demonstrated significantly improved recurrence-free survival in the PCS-TAR cohort, accounting for differences in follow-up duration between groups.

VIHs following OLT represent a distinct and highly complex subset of abdominal wall defects. From an anatomic perspective, these hernias frequently involve intersecting subcostal and upper midline incisions, often span multiple abdominal wall zones, may be multifocal, and are in close proximity to rigid bony structures such as the costal margins and xiphoid process. Repair is further complicated by denervation-related muscle atrophy and, in some cases, loss of domain. Given the technical difficulty of re-entering this operative field, recurrence carries particularly high consequences; in many respects, repair of these hernias represents a “one-shot” opportunity in which durability is paramount.

Patient-specific factors further compound this complexity. Liver transplant recipients are chronically immunosuppressed, frequently have a history of multiple prior abdominal operations, and often present with substantial pre-transplant physiologic burden, as reflected by elevated Model for End-Stage Liver Disease (MELD) scores at the time of transplantation. In addition, the postoperative course following transplantation is often marked by complications and reoperations, which further compromise tissue quality. Beyond patient and anatomic considerations, system-level factors also play a role: access to transplant-experienced surgical teams is not universal, and repair outside the original transplant center may be viewed as high risk by surgeons unfamiliar with the altered anatomy, dense adhesions, and proximity of the transplanted liver.

In the PCS-TAR technique, broad retromuscular mesh overlap provides favorable load distribution across the reconstructed abdominal wall. Reconstitution of the posterior layer creates a continuous, well-vascularized barrier that isolates the mesh from intra-abdominal viscera—not as a primary strength layer, but as a robust biologic floor for the prosthesis. Placement of the mesh within the retromuscular/preperitoneal space effectively situates it between two well-vascularized tissue planes, which may enhance host immune response and the ability to resist or contain infection in an immunosuppressed population. Closure of the anterior fascia, with incorporation of the mesh into the repair, further enhances load sharing and continuity, functioning in effect as a “reverse onlay” configuration. This approach also permits incorporation of distant or satellite defects, such as concomitant umbilical hernias, allowing reconstruction of the entire abdominal wall as a single functional unit.

When viewed in the context of the existing literature, the outcomes observed in the present series are consistent with the heterogeneity reported across prior studies of posterior component separation–based repair in post-transplant patients (Table 6). Published cohorts differ substantially with respect to incision choice, mesh type, mesh position, defect size, and follow-up duration, factors that complicate direct comparison across studies. Notably, series employing a midline incision to address subcostal defects have reported recurrence rates as high as 25%, whereas approaches utilizing the preexisting transplant incision appear to achieve more favorable durability in selected cohorts. This correlates with the Delphi guidelines on subcostal hernias, which recommend avoiding a midline incision unless one is already present [21].

**Table 6:** PCS-TAR repair outcomes in patients with ventral incisional hernias following orthotopic liver transplantation

	CSMC	Tastaldi [13]	Nevo [18]	Miguel-Mendez [19]	Nielsen [20]
Type of repair	PCS - TAR	PCS - TAR	PCS - TAR, ultrapro	PCS - TAR, double mesh	PCS/no TAR, peritoneal flap hernioplasty
Incision	preexisting	midine	midline	preexisting	preexisitng
n (patients)	23	44	10	8	26
Hernia width, cm	10.98	20	8.6	10	8.6
Hernia area, cm²	85	NR	47.9	NR	NR
Mesh area, cm²	253.5	NR	NR	NR	861
Follow-up, months	53	13	25.9	NR	54
LOHS, days,	3.7	7	5.6	6	5.8
*30-day SSI %*	8.6	11.4	7.4	9	0
30-day SSO %	21.7	31.8	42.8	22	3.8
30-day SSOPI %	13	13.6	NR	15	3.8
Hernia recurrence %	0	25	10	2	0

*PCS-TAR* = Posterior Component Separation with Transversus Abdominis Release, *VIHs* = Ventral Incisional Hernias, *LOHS* = Length of Hospital Stay, *SSI* = Surgical Site Infection, *SSO* = Surgical Site Occurrence, *SSOPI* = Surgical Site Occurrence requiring Procedural Intervention, *NR* = not reported

Outcomes reported for alternative repair strategies following liver transplantation further highlight the absence of a uniform standard for managing these complex defects (Table 7). Published series describing preperitoneal, underlay, biologic, bridged, and fascia-based repairs demonstrate substantial variability in recurrence rates, complication profiles, and follow-up duration. Interpretation of these data is limited by heterogeneity in patient selection, operative technique, and reporting of outcomes, as well as by the frequent absence of detailed information regarding defect size, mesh surface area, and abdominal wall reconstruction strategy. Collectively, the literature summarized in Tables 6 and 7 underscores that VIHs following OLT represent a distinct reconstructive challenge for which outcomes are highly sensitive to technical details.

**Table 7:** Outcomes of various repair techniques for ventral incisional hernias following orthotopic liver transplantation

	Ayuso [22]	Shamsaeefar [23]	Heise [24]	Zolper [25]	Perrakis [26]	Justo [27]
Type of repair	preperitoneal, biologic	preperitoneal vs. underlay	underlay composite mesh	underlay biologic/EO release	underlay + onlay	bridged nonvascular fascia
Incision	preexisting	NR	preexisting	preexisting	preexisting	preeexisting
n (patients)	17	60 vs. 28	25	16	29	8
Hernia width, cm	NR	NR	NR	NR	13	12.9
Hernia area, cm²	311	NR	NR	361	NR	92
Mesh area, cm²	818	NR	NR	NR	NR	NR
Follow-up, months	21.6	18+	14.5	36	120	14
LOHS, days,	6.7	NR	NR	NR	6	NR
*30-day SSI %*	5.9	10 vs. 3.6	20	NR	6	0
30-day SSO %	17.6	10 vs. 3.6	28	36.4	24	12.5
30-day SSOPI %	5.9	NR	NR	6.25	NR	12.5
Hernia recurrence %	0	23.3 vs. 17.9	4	6.25	3.4	0

*VIHs* = Ventral Incisional Hernias, *OLT* = Orthotopic Liver Transplantation, *LOHS* = Length of Hospital Stay, *SSI* = Surgical Site Infection, *SSO* = Surgical Site Occurrence, *SSOPI* = Surgical Site Occurrence requiring Procedural Intervention, *NR* = not reported

In the era of minimally invasive repair techniques, the PCS-TAR has been successfully performed laparoscopically and robotically [28]. When applied robotically in ventral hernias, TAR demonstrated shorter LOHS than when open in two meta-analyses and two large registry propensity-matched analyses [29–32]. One randomized trial studying midline hernias verified shorter LOHS of the robotic versus the open group with no difference in composite outcomes [33]. A multi-institutional randomized trial on midline hernias is underway [34]. For subcostal hernias in the post-OLT population, the literature is very limited [35, 36]. Also, it remains unclear whether the above benefits would translate in this population, where dense adhesions, altered anatomy, and proximity to the transplanted liver increase the technical difficulty. A larger study that includes only post-OLT patients and compares open versus robotic technique is necessary.

This study has several limitations. Despite prospective data collection in the PCS-TAR cohort, the OHR group represents a historical comparison, and the study remains retrospective in nature. This design introduces the potential for era-related bias, including changes in perioperative care. Additionally, surgeon experience differed between groups, with PCS-TAR performed by four specialized transplant surgeons compared to fourteen surgeons in the OHR cohort, which may have influenced outcomes. The sample size is modest, and outcomes reflect the experience of a single high-volume transplant center. Furthermore, the heterogeneity of techniques and materials used in the OHR group limits granular comparison, although subgroup analyses were performed to mitigate this and demonstrated consistent findings. Finally, although median follow-up was 53 months in the PCS-TAR cohort and 88.5 months in the OHR cohort, prior literature suggests that complete capture of hernia recurrence may require more than six years of follow-up, and thus late recurrences may be underrepresented in our analysis [37, 38]. Nevertheless, the observed reduction in recurrence and unplanned reoperation following adoption of PCS-TAR represents a clinically meaningful improvement in outcomes for this challenging patient population.

## Conclusion

In OLT recipients with complex VIHs, PCS-TAR performed through the preexisting transplant incision was associated with a markedly reduced recurrence rate and fewer unplanned reoperations, without an increase in short-term morbidity. By prioritizing restoration of abdominal wall anatomy, broad retromuscular mesh overlap, placement of prosthetic material between well-vascularized tissue planes, and isolation of mesh from intra-abdominal viscera, this approach offers a durable and pragmatic solution for a uniquely high-risk patient population. Larger multi-center studies with longer follow-up will be necessary to confirm these findings and to further define optimal reconstructive strategies in post OLT abdominal wall reconstruction.
